# Facts and thoughts on how the COVID-19 pandemic has affected animal agriculture in Argentina

**DOI:** 10.1093/af/vfaa045

**Published:** 2021-02-05

**Authors:** Hugo M Arelovich

**Affiliations:** Departamento de Agronomía, Centro de Recursos Naturales Renovables de la Zona Semiárida (CERZOS), Universidad Nacional del Sur, Bahía Blanca, Argentina

**Keywords:** animal agriculture, Argentina, COVID-19

**Implications**

- On March 20, 2020, a strict nationwide quarantine was announced. The number of infected people and deaths grow daily, and the general impact on welfare and economy is notorious.
- Supply chains have suffered because of national and regional lockdowns, but auctions and direct sales of beef cattle sustain their activities; however, COVID-19 had a negative economic impact on livestock trade fairs and shows.
- Specific protocols have been issued and strictly followed. Thus, the feed industry, slaughterhouses, and transportation have kept providing their services.
- Overall, COVID-19 restrictions per se have not substantially affected animal agriculture in Argentina.

## Sociopolitical and Economic Context in Argentina

Describing Argentina’s political and socioeconomic context in which the COVID-19 pandemic evolved is necessary to weigh the disease’s potential impact on animal agriculture. Argentina is the eighth largest country in the world (2,780,400 km^2^). Presently, the population exceeds 45,000,000 people (National Institute of Statistics and Census of Argentina; INDEC, 2010); however, about 40% of the population is concentrated in Buenos Aires (CABA, 203 km^2^) and its metropolitan area (AMBA, 3833 km^2^). AMBA is a very heterogeneous region with both wealthy and extremely poor large regions. However, CABA assets are political, economic, technological, and cultural power, with minor, but also highly relevant, areas of poverty. CABA was historically the metropolis of Argentina’s middle class. The remaining 60% of the population is unevenly distributed across the 23 provinces of the country with the same problems of middle-class degradation, as well as poverty. In December, 2019 after the national elections, President Mauricio Macri transferred the administration to the elect President Alberto Fernandez (AF).

Regardless of the administration change, Argentina has a structural high inflation rate, increased poverty, and a large external debt. As an example, the inflation rate for 2019 was 53.83% and, up to August 2020, it was 40.67% (INDEC, 2020a), showing no prospective to decrease in the short term. This indicator reflects the fragility of the economy. The impact of the pandemic increased the pressure over the health system and aggravated the economic and social situation.

In this context, on March 20, the government announced the initiation of a strict quarantine to confront COVID-19, even though the outbreak at that time was negligible. There were logical reasons for the decision, such as improving health infrastructure and delaying community spread of the virus. Essential activities were announced and nonessential canceled. These measures were welcome by the citizens for the first 2 months, and President AF’s approval was high. Since then, social fatigue, lack of income, sense of uncertainty, and an inevitable increase in the infection rate and death toll led many to question government mandates related to COVID-19.

Now, 6 months later, schools, research facilities, and universities are still closed, interurban buses and commercial flights never circulated, people can only go to the banks with an appointment, numerous small and large business are broke, the unemployment rate has climbed up, real salary and pensions cannot match up the rise in inflation, and the U.S. dollar exchange rate is increasing permanently in an informal market, while the Central Bank keeps losing its reserves. The government does not seem to have an economic and development program in progress; besides a productive dialogue between officialism and opposition about the real needs of the society does not exist. This situation is increasing social restlessness and deepens the fracture of Argentine society.

The highly populated areas, such as CABA and AMBA, have been the most severely affected initially; however, in the last few weeks, the COVID-19 expanded and increased everywhere. By mid-October 2020, restrictions still continue and Argentina is in the seventh place in the world in COVID-19 total reported cases with a death toll of 2.6% in those infected. The gross national product (GNP) exhibits an interannual variation (August, 2019–2020) of −19.1%, one of the biggest falls in history. Although economic activity in many countries has declined in the second quarter of 2020, Argentina’s situation is more difficult because 25 out of the last 30 months have shown a decrease in the inter-annual GNP.

## Status of Major Animal Commodities

With some exceptions and despite all these burdens, agribusiness seems almost intact, even growing in productivity and trade. This can be attributed to the impetus and positive attitude that characterize this sector—a sector that cannot afford to stop. Fortunately, mostly agribusiness activities are recognized as essential and, thus, exempt from COVID-19-related restrictions on operations and movement. The Ministry of Agriculture, Livestock and Fisheries (MAGYP, 2020a) developed COVID-19 protocols for different agricultural productions.

### Beef industry indicators

**Figure 1.**
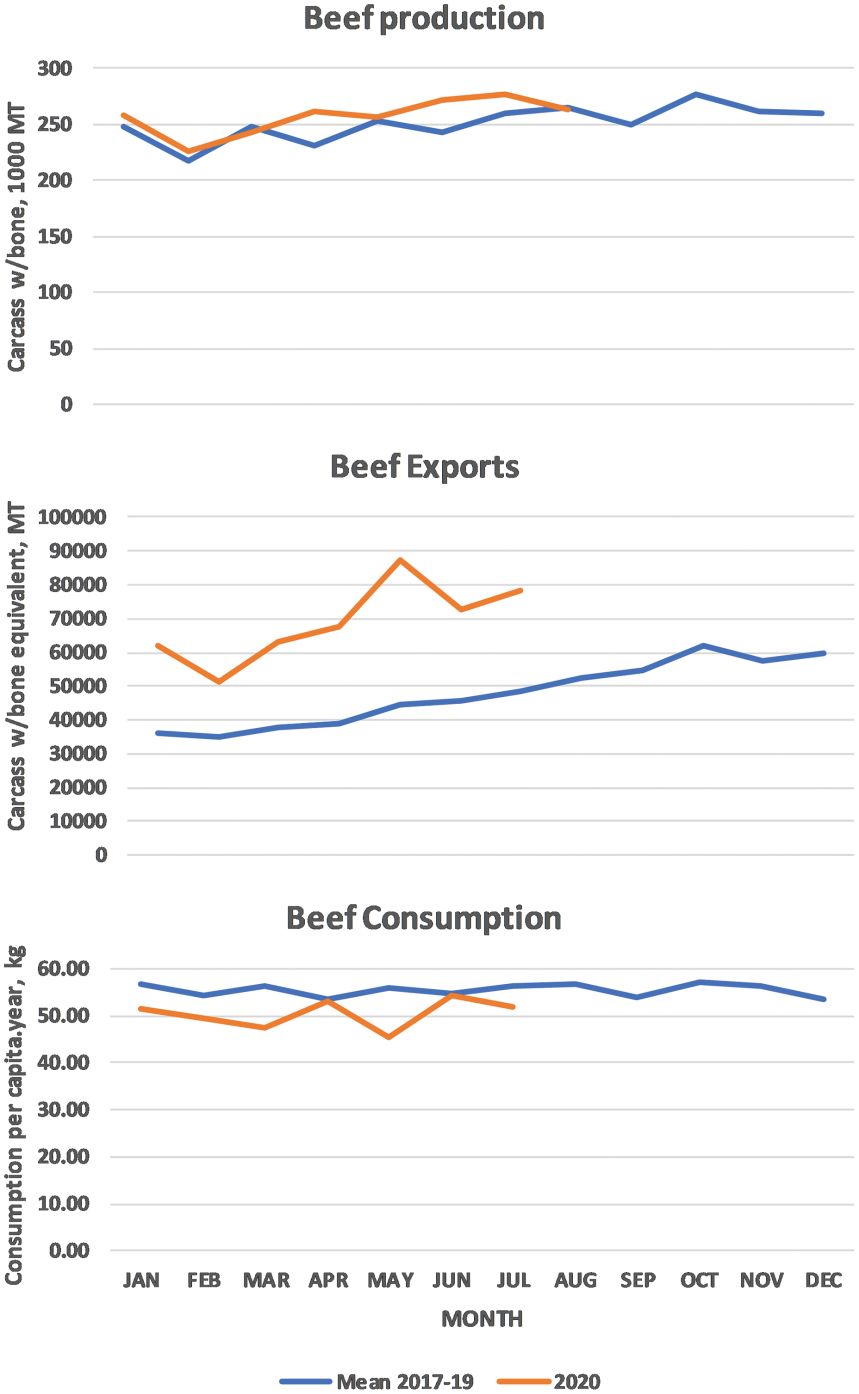
Monthly beef production, exports, and domestic market consumption of averaged periods 2017–2019 and first semester of the year 2020 (elaborated from data MAGYP, 2020b).

The monthly beef production, exports, and domestic market consumption averaged across 2017–2019, compared with the first semester of 2020 (MAGYP, 2020b), are shown in Figure 1. Neither COVID-19 restrictions nor economic context seems to affect beef production or exports. A detailed report from Argentine Beef Promotion Institute (IPCVA, 2020) indicated that the volume of beef production for the second quarter of 2020 shows an interannual expansion of 7.3% compared with the same period in 2019. In the first 5 months of 2020, more than 25% for the beef demand was from China but, in June, it fell below 20%; this makes it difficult to predict exports volume. However, the meat that was not exported to China was compensated by a considerable purchase from the United States.

Beef cattle, with a stock of more than 54 million heads, is the most relevant animal product in Argentina due to cultural significance and eating habits. When affordable, consumers prefer beef over other meats. In Figure 1, we can observe a decrease in beef consumption per capita for the first semester of 2020. This can be attributed to an increase in retail prices, low incomes, and job loss, undoubtedly aggravated by the COVID-19 pandemic. Retail prices, as of June 2020, grew at a year-on-year rate of 52.6%, significantly above the general inflation rate of around 42.8%. Still, 74% of the beef produced supplies the domestic market, and the remaining 26% go to exports (IPCVA, 2020). Argentina’s total exports for the first semester of 2020 reached 27.336 million USD, with a great collapse in the interannual variation of the oil-petrochemical and automotive sectors, among others (INDEC, 2020b).

However, exports in U.S. dollars for the main agribusiness sector (soybeans, corn, and wheat), with a proportional participation of 21.5%, showed a positive interannual variation. Beef and leather made up a significant 5.9% share of U.S. dollars income. Despite the increase in metric tons (MT) exported compared to 2019, in US dollars, beef had a low interannual variation of −3.9% probably because international prices plunged 23%.

### Other meat products indicators

**Figure 2.**
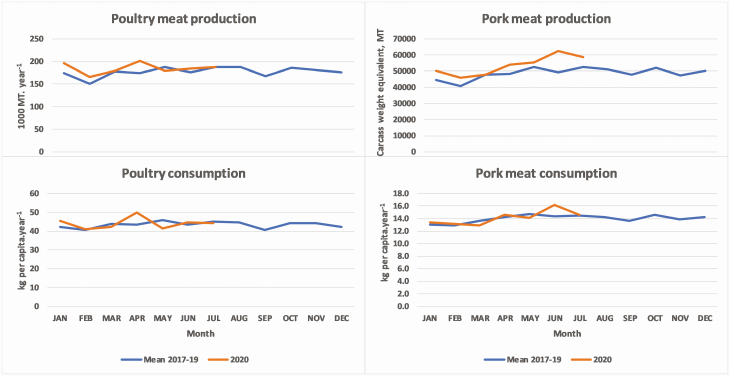
Monthly poultry and pork production and domestic market consumption of averaged periods 2017–2019 and first semester of the year 2020 (elaborated from data MAGYP, 2020c,d).

Argentina’s “protein basket” per capita is made up of 50.9 kg of beef, 43.9 kg poultry, and 14.5 kg pork for a total 108.4 kg per year of a variety meats (Brusca, 2020). Although, in Argentina, there is about 15 million head of sheep, their meat consumption is negligible; some estimates indicate only about 1 kg per capita annually. Poultry and pork production and domestic market consumption average across 2017–2019 and the first semester of 2020 (MAGYP, 2020c,d) are shown in Figure 2. Reported interannual variation (January–July, 2019–2020) indicates that production was +2.3% and +5.5%, consumption +2.9% and −1.3%, and exports −1.3% and +66% for poultry and pork meat, respectively. It looks like the increase in poultry meat accounted for the decrease in beef consumption. The dramatic increase in exports from the pork industry was mainly because of Chinese demands, but the increase also shows the growth of this industry in Argentina. Currently, the African Swine Fever outbreak in Europe is an unfortunate fact; nevertheless, it can open a new business opportunity for the Argentine pork industry. Consequently, up to date, COVID-19 restrictions do not seem to affect the variables presented here for the poultry and pork industry.

### Dairy industry indicators

**Figure 3.**
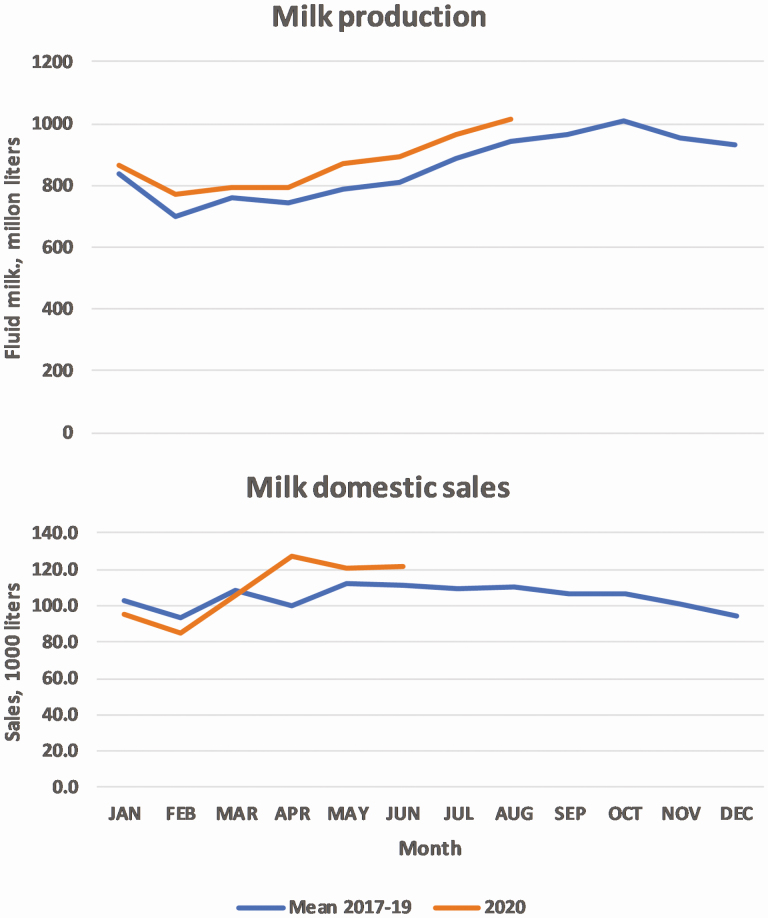
Monthly fluid milk production and domestic market sale, considered as equivalent to the consumption of averaged periods 2017–2019 and first semester of year 2020 (elaborated from data MAGYP, 2020e). Other dairy products are not included in this report.

Fluid milk production and consumption exhibited an almost sustained fall since 2010, parallel with the drop of GNP per capita (−17%) up to 2019. A moderate but sustained increase in fluid milk production for the first semester of 2020 compared to the mean of the period 2017–2020 can be seen in Figure 3, as well as April–May peak for total sales (MAGYP, 2020e).

Numerous other products, such as yoghurts and cheeses, are produced by the dairy industry in Argentina, but those more expensive and, unfortunately, might not be as accessible to many people with the lowest income. Compared to the first half of 2019, exports of powdered milk and cheese were 63,809 and 19,707 MT, a total of + 51.3% and −24.6%. Different variables, other than COVID-19, seem to have influenced production and export fluctuations within the dairy industry.

## Supply Chains

From the very beginning of the pandemic, the circulation of people was limited, but essential supplies were somewhat guaranteed. As in other parts of the world, more and more activities have been allowed as the pandemic has continued. However, businesses did not open up evenly in the country, and there are differences in the duration and strictness of the protocols— and bottlenecks—because of national and regional (provinces) lockdowns. Fernando Canosa (Livestock Consulting at GANADERO, professional service, personal communication, 2020), a nationally recognized beef cattle consultant, stated “…our livestock sector is actively working, the animals continue to produce, gestate and calving, and those who have to re-breed continue to do so. What worries are the province closures, with permits that are often useless. This is a serious threat to production by preventing the transfer of inputs, products, contractors, professionals and entrepreneurs, etc. If the situation is not resolved quickly, future production is at stake.”

## Animal Farming and Trade

Except for inconveniences in obtaining some specific supplies or social distancing, no reports indicate that COVID-19 has affected operations inside farms. A national survey among members of the Regional Consortium for Agricultural Experimentation (CREA, 2020), a group for agricultural entrepreneurs, indicated that around 15% of members had difficulties commercializing their production because of COVID-19 restrictions, and this ranged between 7% and 29% of members depending on their type of production and area of the country. A clear majority (85%) said that they were “unchanged.”

In general, regular commercial auctions and direct sales of beef cattle sustain the flux of heads to maintain the dynamics of production systems and the supply of meat. Online auctions have also been held for years. For in-person auctions, specific protocols have to be followed for those with attendance. As an example, the Ministry of Agrarian Development of the Province of Buenos Aires (MDA, 2020) approved specific protocols for cleaning and disinfection, and it made recommendations for the transport, loading and unloading of animals, and in the physical space of the fair auction (MDA, 2020).

Despite these measures, COVID-19 has had a very negative economic and social impact on livestock trade fairs and shows, which are of economic relevance and very popular around the country. The Palermo Rural Exposition at CABA, the most important show in Argentina that attracts thousands of people every year, was suspended before its 134th edition and rescheduled for 2021. The 136th National Exposition of Livestock, at Bahía Blanca, and other traditional annual exhibitions were conducted on schedule but without the public. At Bahía Blanca, exposition was restricted to beef cattle only and held without the industrial and commercial exhibit; however, the shows could be followed through online streaming. Some other customary shows at a smaller scale were either conducted with limitations or suspended according to diverse criteria and regional COVID-19 trends.

The feed industry was also declared essential. According to Cecilia Inchausti (CEO at CRECER FEEDS, Tornquist, Argentina, personal communication, 2020), a regional leader in feed manufacturing, they suffered delays the first 2 wk after national quarantine was initiated; thereafter, permits were issued and transportation became more regular, at least in the areas they cover with their business. They also adopted precise work protocols to protect their operators, as well as to ensure continuous production. They are working in teams that do not cross each other and, if they have a COVID-19 case, the whole team is isolated and the other team takes over the job. Most of the problems they face are more related to the economic and financial uncertainty than to the pandemic itself. Inchausti believes that these facts are true for most of the feed industry.

## Slaughterhouses: Outbreaks of COVID-19

The evolution of the COVID-19 pandemic is of great concern for slaughter houses and packing plants, which are in high alert for COVID-19 around the globe. In Argentina, a strict protocol was issued by SENASA (MAGYP, 2020a) and is applied at all plants. Only two plants were suspended by mid-September 2020, and there were six plants that temporarily self-excluded due to COVID-19 cases among employees. Four of these plants are for poultry, three for beef, and one for pork. So far, very few plants have been affected, but some people expect that the number of workers infected may rise as the virus advances.

Although there is no evidence that COVID-19 can be transmitted through food, slaughterhouses became a real bottleneck because the workers are at a high risk of being infected. Besides the concern for their health, difficulties marketing products, worker absence, and sanitary controls could slow or stop production.

## Education, Research, and Extension

All educational institutions, from kindergartens to universities, have been closed for the last 6 months. The National Institute of Agricultural Technology has research stations all along the country; an institutional protocol has affected sites to different extents. Their extension activities are being intensively carried out online. Universities stay closed and activities are mostly reduced to teaching online. Overall, on-going projects are allowed to proceed with specific permits, but new projects have been temporarily canceled, particularly those that require the interaction of multiple individuals and intensive lab activity.

However, commercial labs remain productive. For example, the U.S. Department of Agriculture has established an agreement with an Argentine pharmaceutical company to be one of its suppliers of antigens and vaccines for a new bank created to reinforce protection measures against foot-and-mouth disease (Valor Carne, 2020).

## Final Thoughts

So far, COVID-19 restrictions per se have not substantially affected animal agriculture in Argentina, but restrictions have changed our sociopolitical and economic situation, which, in turn, has somewhat impacted productivity and trade variables. However, different results could be seen in the next months or year since the pandemic is highly dynamic. Our expectations for the future can be defined by one word: “uncertain.”

Although the country’s poverty rate increased to 40.9% of the population—and we have seen the impact of COVID-19 on wages and job loss—the consumption of animal products (except for milk) seems to be stable. It makes sense that some consumption is sustained by the portion of the population that maintains purchasing power, plus government emergency assistance programs, which were added to the many already existing (INDEC, 2020c).

*Conflict of interest statement*. None declared.

## About the Author

**Hugo M. Arelovich** received a diploma of Ingeniero Agrónomo from Universidad Nacional del Sur (UNS), Argentina in 1976, and completed graduate degrees at Oklahoma State University (OSU), Master of Science (1983) and PhD (1998). In 2016, he received the *Advanced Degree Graduate of Distinction Award* from OSU. As a faculty member at UNS, retired in 2017 after 39 years of service, following recognition by his institution as “*Profesor Extraordinario Consulto,*” which allows him to keep office and stay academically active. His job duties included undergraduate and graduate teaching in animal nutrition and research in ruminant nutrition. He was President of the Argentinian Association of Animal Production in 2009–2011, and 2017–2019. He participated in invited talks and other numerous activities related with professional organizations, producers, and academics in Argentina, United States, Canada, Panama, Brazil and Uruguay. He also provides consultancy services to producers, professionals, and feed industry.
